# Angiotensin-(1-7) and Alamandine Promote Anti-inflammatory Response in Macrophages *In Vitro* and *In Vivo*

**DOI:** 10.1155/2019/2401081

**Published:** 2019-02-21

**Authors:** Melissa de Carvalho Santuchi, Miriane Fernandes Dutra, Juliana Priscila Vago, Kátia Maciel Lima, Izabela Galvão, Fernando Pedro de Souza-Neto, Mario Morais e Silva, Aline Cristina Oliveira, Flávia Carvalho Bittencourt de Oliveira, Ricardo Gonçalves, Mauro Martins Teixeira, Lirlândia Pires Sousa, Robson Augusto Souza dos Santos, Rafaela Fernandes da Silva

**Affiliations:** ^1^Department of Physiology and Biophysics, Institute of Biological Sciences, Federal University of Minas Gerais, Belo Horizonte, Brazil; ^2^Department of Clinical and Toxicological Analyses, Faculty of Pharmacy, Federal University of Minas Gerais, Belo Horizonte, Brazil; ^3^Department of Biochemistry and Immunology, Institute of Biological Sciences, Federal University of Minas Gerais, Belo Horizonte, Brazil; ^4^Department of Pathology, Institute of Biological Sciences, Federal University of Minas Gerais, Belo Horizonte, Brazil

## Abstract

The renin-angiotensin system (RAS) peptides play an important role in inflammation. Resolution of inflammation contributes to restore tissue homeostasis, and it is characterized by neutrophil apoptosis and their subsequent removal by macrophages, which are remarkable plastic cells involved in the pathophysiology of diverse inflammatory diseases. However, the effects of RAS peptides on different macrophage phenotypes are still emerging. Here, we evaluated the effects of angiotensin-(1-7) (Ang-(1-7)) and the most novel RAS peptide, alamandine, on resting (M0), proinflammatory M(LPS+IFN-*γ*), and anti-inflammatory M(IL-4) macrophage phenotypes *in vitro*, as well as on specific immune cell populations and macrophage subsets into the pleural cavity of LPS-induced pleurisy in mice. Our results showed that Ang-(1-7) and alamandine, through Mas and MrgD receptors, respectively, do not affect M0 macrophages but reduce the proinflammatory TNF-*α*, CCL2, and IL-1*β* transcript expression levels in LPS+IFN-*γ*-stimulated macrophages. Therapeutic administration of these peptides in LPS-induced inflammation in mice decreased the number of neutrophils and M1 (F4/80^low^Gr1^+^CD11b^med^) macrophage frequency without affecting the other investigated macrophage subsets. Our data suggested that both Ang-(1-7) and alamandine, through their respective receptors Mas and MrgD, promote an anti-inflammatory reprogramming of M(LPS+IFN-*γ*)/M1 macrophages under inflammatory circumstances and potentiate the reprogramming induced by IL-4. In conclusion, our work sheds light on the emerging proresolving properties of Ang-(1-7) and alamandine, opening new avenues for the treatment of inflammatory diseases.

## 1. Introduction

The renin-angiotensin system (RAS) is composed of multiple enzymes and small effector peptides involved in blood pressure regulation and hydroelectrolytic balance [1]. Beyond these classical functions, there are a growing number of studies revealing the involvement of RAS components in inflammation [2–7]. While it is well established that angiotensin (Ang) II elicits proinflammatory properties in a range of target organs [7, 8], angiotensin-(1-7) (Ang-(1-7)), a heptapeptide derived from Ang I and Ang II, is able to counter-regulate these effects [2, 6, 9]. Indeed, through the activation of the Mas receptor, Ang-(1-7) has been shown to inhibit or attenuate the inflammatory process in distinct experimental models of inflammatory diseases, including obstructive sleep apnea, chronic pulmonary allergy, atherosclerosis, and sepsis [3, 6, 10–12]. Interestingly, Mas receptor deficiency exacerbated the course of experimental autoimmune encephalomyelitis in mice and increased macrophage infiltration, as well as Th1 cell frequency in the central nervous system [4]. Moreover, Barroso et al. have recently reported that Ang-(1-7), through the Mas receptor, enhances both neutrophil apoptosis and its removal by macrophages (efferocytosis) in an animal model of arthritis [6].

Macrophages are key players of tissue homeostasis [13–15]. *In vitro*, resting macrophages (M0) have the potential to dynamically change their secretory and functional profile, in response to a switch in their milieu, towards at least three subsets: classically activated macrophages, anti-inflammatory/wound healing macrophages, and regulatory macrophages [16]. *In vivo*, the complex dynamics underlying each disease lead to such an environment-dependent cell response that it is difficult to delineate each macrophage subset [17, 18]. However, *in vitro* macrophages can be polarized in a highly controllable manner, which allows the induction of very different and specific phenotypes. Macrophages treated with lipopolysaccharides and interferon-*γ* (M(LPS+IFN-*γ*) macrophages) or interleukin-4 (M(IL-4) macrophages) can be polarized toward pro- and anti-inflammatory profiles, respectively [16]. Nevertheless, there are only a few studies addressing the effects of Ang-(1-7)/Mas receptor activation on macrophage functions. Souza and Costa-Neto et al. demonstrated that Ang-(1-7) reduces the LPS-induced mRNA expression of tumor necrosis factor-*α* (TNF-*α*) and IL-6 in mouse peritoneal macrophages [19]. More recently, Hammer and colleagues showed that the deletion of the Mas receptor in mice differentially regulates the expression of pro- and anti-inflammatory transcripts in macrophages exposed to either LPS+IFN-*γ* or IL-4+IL-13, respectively [4]. These studies reinforce the anti-inflammatory properties of the activation of this protective arm from the RAS. However, data on the relationship of RAS peptides and M(IL-4) macrophages in inflammation are scarce and often contradictory [4, 20].

A new RAS peptide, Ala^1^-(Ang-(1-7)) (alamandine), has been recently described by our collaborators in 2013 [21]. Alamandine can be generated by the enzymatic decarboxylation of Ang-(1-7), by a not yet known enzyme, or by ACE2-mediated cleavage of Ang A [21]. Interestingly, the protective actions of this peptide in the cardiovascular system were not inhibited by the classical Mas receptor antagonist, A-779, but by D-Pro^7^-Ang-(1-7) (D-Pro^7^), which binds to Mas-related G protein-coupled receptor member D (MrgD) [21]. The structural difference between these two peptides is the amino acid Asp in the N-terminal position of Ang-(1-7) sequence that is replaced by Ala in alamandine. There are a growing number of studies investigating the cardiovascular effects of alamandine/MrgD receptor activation; however, little is known about its anti-inflammatory potential [22]. In the present study, we have investigated the *in vitro* effects of Ang-(1-7) and alamandine on M0, M(LPS+IFN-*γ*)-, or M(IL-4)-stimulated macrophages and the effects of these peptides on leukocytes and macrophages subsets using a murine model of LPS-induced pleurisy.

## 2. Materials and Methods

### 2.1. Animals

FVB/N and BALB/c male mice (8-10 weeks) were obtained from laboratory animal facilities from the Institute of Biological Sciences of the Federal University of Minas Gerais (ICB/UFMG). All *in vitro* experiments were performed on macrophages obtained from FVB/N mouse lineage. The model of LPS-induced pleurisy was performed in BALB/c mice as previously standardized [23]. The animals were housed under standard conditions and had access to water and chow *ad libitum* until the end of the experimental protocols. All animal care and experimental procedures were performed under previously approved protocols by the Animal Ethics Committee of the Federal University of Minas Gerais (protocols 340/2016 and 183/2017).

### 2.2. Isolation and Characterization of Bone Marrow-Derived Macrophages

The animals were anesthetized in a chamber with isoflurane (3%) prior to cervical dislocation. Bone marrow cells were isolated and cultured until differentiation into macrophages for 8 days as recommended by Mosser and Gonçalves [24]. Briefly, bone marrow cells were harvested from tibias and femurs of FVB/N mice and cultured in macrophage complete medium supplemented with 20% of centrifuged and filtered L929 cell supernatant media, under standard conditions (5% CO_2_ and 37°C in incubators). After 72 hours, the medium was washed and replaced by a new fresh medium to allow cell differentiation towards bone marrow-derived macrophages (BMDM). On day 8, the medium was discarded and the cells were washed one time with phosphate-buffered saline (PBS) before the *in vitro* stimulation protocols. The 4-hour *in vitro* polarization towards inflammatory phenotype M(LPS+IFN-*γ*) (using LPS at 1 *μ*g/mL+IFN-*γ* at 20 ng/mL) and anti-inflammatory M(IL-4) macrophages (using IL-4 at 20 ng/mL) was performed according to Pelegrin and Surprenant [25]. Total ribonucleic acid (RNA) was extracted with TRIzol (Invitrogen, USA) and purified with DNAseI Amplification Grade (Sigma, USA) before quantification using a spectrophotometer (NanoDrop® ND-1000, Thermo Scientific, USA). For reverse transcription polymerase chain reaction (PCR), 2 micrograms of RNA was used following all manufacturers' specifications for MMLV reverse transcriptase (Invitrogen, USA). The expression levels of a set of transcripts for M(LPS+IFN-*γ*) macrophages: TNF-*α*, chemokine (C-C motif) ligand 2 (CCL2), and interleukin-1*β* (IL-1*β*); and M(IL-4) macrophages: chitinase 3-like (YM1), found in inflammatory zone 1 protein (FIZZ1) and mannose receptor type C1 (MRC1) markers were analyzed by quantitative real-time polymerase chain reaction (qPCR) using Maxima™ Rox SYBR Green qPCR Master Mix (Thermo Fisher Scientific, USA), glyceraldehyde 3-phosphate dehydrogenase (GAPDH) as endogenous control, and M0 as the reference sample. The messenger RNA (mRNA) expression levels of Mas and MrgD receptors in M0, M(LPS+IFN-*γ*), and M(IL-4) macrophages were also evaluated. Primer sequences are listed in Table S1 (Supplementary Material).

### 2.3. Immunofluorescence for Mas and MrgD Receptors in Macrophages

The protein expression and cellular localization of Mas and MrgD receptors in the M0, M(LPS+IFN-*γ*), and M(IL-4) groups were evaluated by immunofluorescence. After 4 h of *in vitro* stimulation, cells were washed with PBS 1x and fixed for 15 minutes at room temperature with 4% paraformaldehyde (PFA). Cell membranes were permeabilized with a solution of 0.5% Triton X-100/PBS for 10 minutes at room temperature, and then, samples were incubated with 5% bovine serum albumin (BSA)/Tween-20/PBS solution for 1 hour at room temperature. Samples from all groups were incubated with anti-rabbit polyclonal primary antibody to the Mas receptor (Alomone Labs, USA) [26] or to the MrgD receptor (Abcam, United Kingdom) [27] at 1 : 300 blocking solution, overnight at 4°C. After washing with PBS 1x, coverslips containing cells were incubated with goat anti-rabbit oligoclonal secondary antibody conjugated to Alexa Fluor® 555 (1 : 450, Molecular Probes, USA) for the detection of the Mas receptor or Alexa Fluor® 488 receptor (1 : 300, Molecular Probes, USA) for MrgD in PBS for 1 h. Thereafter, the slides were incubated with 4′,6-diamidino-2-phenylindole (DAPI) (1 : 150, Molecular Probes, USA) and mounted on Fluoromount G (SouthernBiotech, USA). Microscopy analysis was immediately performed with excitation at 488 nm and 560 nm for MrgD and Mas receptors, respectively. Negative controls for all immunofluorescent staining experiments were performed. For that purpose, we have followed identical staining protocols, however, without adding the primary antibodies. The cellular immunofluorescence signals were analyzed under an Axio Imager Apotome.2 microscope (Zeiss, Germany) with 40x magnification immersion oil objective lens. The images were captured with an AxioCam 503 Color camera (Zeiss, Germany) and analyzed in the Fiji program [28], which allowed the collection of the area (*μ*m^2^) and integrated fluorescence intensity data of 42-50 cells/*n*/group.

### 2.4. *In Vitro* Treatment of Macrophages with Ang-(1-7) or Alamandine

The concentration of Ang-(1-7) and alamandine (10^−7^ M) used in the present study was based on previous works in experimental models of inflammation [21, 29–31]. Ang-(1-7) and alamandine were obtained from Biosyntan (Germany) and Bachem (Switzerland), respectively.

To evaluate the ability of Ang-(1-7) and alamandine to reprogram M0 macrophages, we first measured the expressions of M(LPS+IFN-*γ*) and M(IL-4) specific transcripts in M0 macrophages *in vitro*. In order to assess the effects of Ang-(1-7) and alamandine on M(LPS+IFN-*γ*) or M(IL-4) phenotypes, the cells were pretreated with either Ang-(1-7), alamandine, or vehicle (distilled water) in culture medium by thirty minutes after the beginning of the polarization until its end (4 h later). qPCR was used to determine the expression of the following M(LPS+IFN-*γ*) transcript markers: CCL2, IL-1*β*, and TNF-*α*, and M(IL-4) transcript markers: YM1, FIZZ1, and MRC1. We further analyzed the expression of MRC1, arginase-1, and iNOS mRNAs after 24-hour treatment with the peptides *in vitro* to understand the effects of a long-term exposition of M0 macrophages to Ang-(1-7) and alamandine. To evaluate the role of the Ang-(1-7)/Mas receptor and alamandine/MrgD receptor pathways on the observed effects, we used their pharmacological antagonists (D-Ala^7^)-Ang I/II (1-7) (A-779) (Biosyntan, Germany) and D-Pro^7^ (Bachem, Switzerland), respectively. Cells were treated with A-779 (10^−6^ M) or D-Pro^7^ (10^−6^ M) for twenty-five minutes before the administration of Ang-(1-7) or alamandine, respectively. Furthermore, to investigate cell plasticity, M(LPS+IFN-*γ*) macrophages were treated with Ang-(1-7) or alamandine as mentioned. The expression of transcripts for M(IL-4) markers was analyzed by qPCR.

To understand the relationship among Ang-(1-7) and alamandine concentrations and the cell response, different peptide concentrations (10^−9^, 10^−8^, and 10^−7^ M) were used. The mRNA expressions of TNF-*α* and YM1 in M(LPS+IFN-*γ*) and M(IL-4) macrophages, respectively, were evaluated by qPCR as previously mentioned.

### 2.5. The Effects of Ang-(1-7) and Alamandine on Leukocyte Population and Macrophage Subsets on LPS-Induced Pleurisy in Mice

BALB/c mice received an intrapleural (i.pl.) injection of LPS (250 ng/cavity) from *Escherichia coli* (serotype O:111: B4, Sigma-Aldrich, USA) or PBS as previously described [23, 32, 33]. Eight hours after (at the expected peak of neutrophilic infiltration), LPS-injected mice were randomly divided into 4 groups to evaluate the effects of Ang-(1-7) and alamandine on pleurisy: PBS only (*n* = 6 animals), LPS only (*n* = 7 animals), LPS+Ang-(1-7) (*n* = 8 animals), and LPS+alamandine (*n* = 8 animals). The animals were treated with either a single dosage of 45 *μ*g·kg^−1^ Ang-(1-7) or alamandine in 2-hydroxypropyl-*β*-cyclodextrin (HP*β*CD, vehicle) by gavage [9, 21]. After 24 h, the cells present in the pleural cavity were harvested by washing the cavity with 2 mL PBS. Total cell counts were performed in a Neubauer chamber using Turk's stain. Differential cell counts were performed on cytocentrifuge preparations (Shandon III) stained with May-Grunwald-Giemsa using standard morphological criteria to identify cell types as published [23, 34–36]. The populations of neutrophils and macrophages were also evaluated by flow cytometry. Macrophages were F4/80^+^CD11b^+^, and neutrophils were F4/80^−^GR1^+^. The results are presented as the number of cells per cavity or the percentage of the total number of cells.

In order to determine the macrophage subsets into the pleural cavity, we have used a previously published protocol based on the established markers of 3 subpopulations of macrophages: M1 macrophages (F4/80^low^Gr1^+^Cd11b^med^), M2 macrophages (F4/80^high^Gr1^–^Cd11b^high^), and resolution-promoting macrophages (Mres) (F4/80^med^Cd11b^low^) by flow cytometry [23, 37, 38]. Samples were stained with fluorescent monoclonal antibodies against F4/80 (PE-Cy7, clone BM8, eBioscience, USA), Gr1 (FITC, clone RB6-8C5, BioLegend, USA), and CD11b (PE-Cy5, clone M1/70, BD Biosciences, USA) and then acquired in a BD LSRFortessa cell analyzer (BD Biosciences). These samples were analyzed using FlowJo software (Tree Star, USA). Gating strategy was conducted as previously described [23], and it is available in the supplement material (Figure S3). Unstained cells were used as negative controls. The results are presented as the frequencies for each macrophage subset.

### 2.6. Statistical Analysis

The analysis of the data was performed by Student's *t*-test or one-way ANOVA followed by the Newman-Keuls posttest for normally distributed or log-transformed data. The Kruskal-Wallis or Mann-Whitney test followed by Bonferroni posttest was used for nonnormally distributed data. For the *in vitro* experiments, we have performed *n* = 4 and in four replicates. The *in vivo* experiment was repeated three times, resulting in similar findings. Data are expressed as the means ± standard error mean (SEM), and statistical differences were considered when *P* < 0.05. All graphics and analysis were made with the Past software (Natural History Museum, University of Oslo, Norway).

## 3. Results

### 3.1. *In Vitro* Characterization of the Transcriptional Profile of Mas and MrgD Receptor Expressions in Macrophages

**Figure 1:**
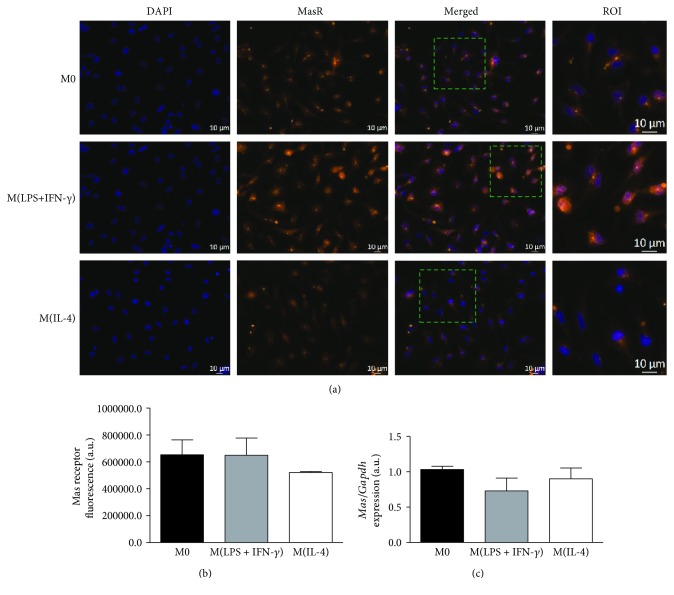
*In vitro* expression of the Mas receptor in M0, M(LPS+IFN-*γ*), and M(IL-4) macrophages. (a) Representative images of the Mas receptor detection by immunofluorescence (orange) and nuclei by DAPI (blue). (b) Quantification of fluorescence intensity and (c) relative mRNA expression for the Mas receptor normalized by the M0 group in M(LPS+IFN-*γ*) and M(IL-4) phenotypes. Results were obtained by one-way ANOVA (1b) or by the nonparametric Kruskal-Wallis test (1c) and are expressed as the mean ± SEM of *n* = 3 − 4 and *n* = 4 independent experiments for protein and mRNA, respectively.

**Figure 2:**
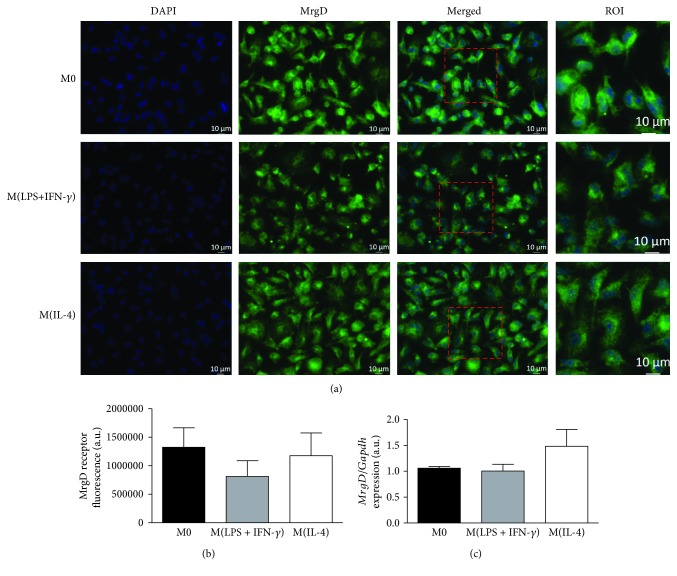
Expression of the MrgD receptor in M0, M(LPS+IFN-*γ*), and M(IL-4) macrophages *in vitro*. (a) Representative images of the MrgD receptor detection by immunofluorescence (green) and nuclei by DAPI (blue). (b) Quantification of fluorescence intensity and (c) relative mRNA expression for MrgD normalized by the M0 group in M(LPS+IFN-*γ*) and M(IL-4) phenotypes. Results were obtained by one-way ANOVA (2b) or by the nonparametric Kruskal-Wallis test (2c) and are expressed as the mean ± SEM of *n* = 4 independent experiments for both protein and mRNA.

The characterization of M0, M(LPS+IFN-*γ*), and M(IL-4) macrophage phenotypes was performed by analyzing their differential mRNA expression for TNF-*α*, CCL2, IL-1*β*, YM1, FIZZ1, and MRC1 (Figure S1). The expression and pattern of distribution of Mas and MrgD receptors at both mRNA and protein levels were evaluated by qPCR and immunostaining, respectively. The expression of the Mas receptor showed a diffuse distribution throughout the cell extension in the M0, M(LPS+IFN-*γ*), and M(IL-4) groups, with no differences in protein or mRNA expression levels among them (Figures 1(a)–1(c)). A similar diffuse cellular distribution pattern was observed for the staining of the MrgD receptor, with no significant differences in protein or transcript expression (Figures 2(a)–2(c)). These results suggest that macrophage polarization does not seem to affect the expression of the Mas and MrgD receptors in our *in vitro* model, at the analyzed time point of 4 h.

### 3.2. Ang-(1-7) and Alamandine Differentially Affect Macrophage Subsets and Their *In Vitro* Inflammatory Responses

**Figure 3:**
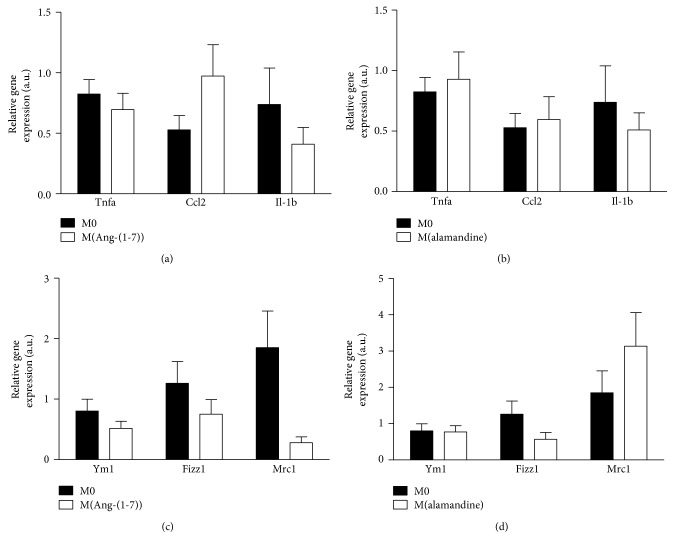
Expression profile for M(LPS+IFN-*γ*) and M(IL-4) markers in M0 before and after the treatment with Ang-(1-7) or alamandine. (a, b) Real-time PCR analysis of TNF-*α*, CCL2, and IL-1*β* levels in M0 macrophages before and after treatment with Ang-(1-7) (10^−7^ M) (a) or alamandine (10^−7^ M) (b). (c, d) Real-time PCR analysis of YM1, FIZZ1, and MRC1 transcripts expressions in M0 macrophages before and after treatment with Ang-(1-7, 10^−7^ M) (c) or alamandine (10^−7^ M) (d). Results were obtained by a *t*-test or nonparametric Mann-Whitney test and are expressed as the mean ± SEM of *n* = 4 independent experiments.

**Figure 4:**
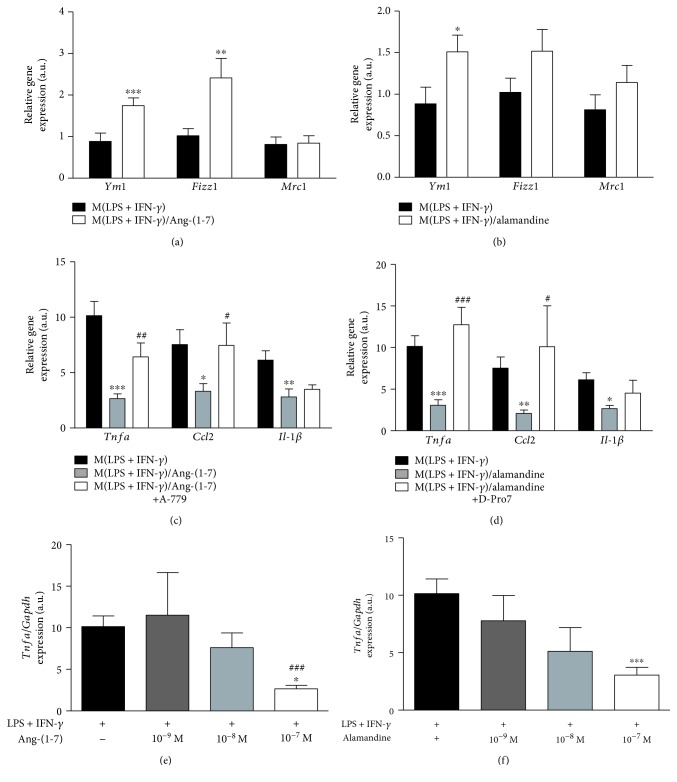
*In vitro* Ang-(1-7)- and alamandine-induced anti-inflammatory response and cell plasticity in M(LPS+IFN-*γ*) macrophages. Real-time PCR analysis of YM1, FIZZ1, and MRC1 [M(IL-4) macrophage markers] in M(LPS+IFN-*γ*) macrophages after treatment with Ang-(1-7) (10^−7^ M) (a) or alamandine (10^−7^ M) (b), evidencing cell plasticity. (c) Mas receptor-mediated anti-inflammatory response in M(LPS+IFN-*γ*) macrophages analyzed by real-time PCR for TNF-*α*, CCL2, and IL-1*β* before and after treatment with Ang-(1-7) or Ang-(1-7)+A779 (A779; 10^−6^ M). (d) MrgD receptor-mediated anti-inflammatory response in M (LPS+IFN-*γ*) macrophages for TNF-*α*, CCL2, and IL-1*β* before and after treatment with alamandine or alamandine+D-Pro^7^ (D-Pro^7^; 10^−6^ M). (e) Ang-(1-7) and (f) alamandine concentration-dependent TNF-*α* mRNA levels in M(LPS+IFN-*γ*) macrophages. Results were obtained by a *t*-test and nonparametric Mann-Whitney test for comparisons between two groups (a and b) or one-way ANOVA and nonparametric Kruskal-Wallis test for comparisons among three or more groups (c–f) and are expressed as the mean ± SEM of *n* = 4 independent experiments. ^∗∗∗,###^ *P* < 0.001, compared to control and to peptide treatment, respectively; ^∗∗,##^ *P* < 0.01, compared to control and peptide treatment only; ^∗,#^ *P* < 0.05, compared to control and peptide treatment only, respectively.

Next, we evaluated whether the peptides per se could reprogram macrophages. Thus, M0 cells were treated with either Ang-(1-7) or alamandine (10^−7^ M) and the expression of the major pro- and anti-inflammatory transcript markers was measured by qPCR. None of the peptides affected the expression levels of TNF-*α*, CCL2, and IL-1*β* (Figures 3(a) and 3(b)), as well as YM1, FIZZ1, and MRC1 transcripts (Figures 3(c) and 3(d)). Accordingly, a 24-hour *in vitro* peptide treatment did not affect the expression of iNOS, MRC1, and arginase-1 in M0 macrophages (Figure S2). The analysis of the mRNA expression for M(IL-4) markers in the M(LPS+IFN-*γ*) macrophage subset *in vitro* showed that both Ang-(1-7) and alamandine increased YM1 expression, and Ang-(1-7) was also able to induce FIZZ1, but no difference was observed for MRC1 levels (Figures 4(a) and 4(b)). These results were consistent with the decrease in the expression of the proinflammatory TNF-*α*, CCL2, and IL-1*β* mRNA observed following the treatment of M(LPS+IFN-*γ*) macrophages with Ang-(1-7) (Figure 4(c)). To evaluate the role of the Mas receptor on the anti-inflammatory responses evoked by Ang-(1-7) in M(LPS+IFN-*γ*) macrophages, we treated the cells with a Mas receptor antagonist, A-779, prior to Ang-(1-7) incubation. A-779 totally reversed the effects of Ang-(1-7) on TNF-*α* and CCL2 but did not alter IL-1*β* mRNA expression in M(LPS+IFN-*γ*) macrophages (Figure 4(c)). The treatment with alamandine evoked a similar response in M(LPS+IFN-*γ*) macrophages as did Ang-(1-7). As observed in Figure 4(d), alamandine was able to decrease the expression levels of all three inflammatory markers. In addition, we evaluated the effect of alamandine on the MrgD receptor in M(LPS+IFN-*γ*) macrophages after treatment with a MrgD receptor antagonist, D-Pro^7^. In a similar way to the Ang-(1-7) antagonist, MrgD receptor pharmacological antagonism only reversed the expression of TNF-*α* and CCL2 mRNA levels (Figure 4(d)). The inhibitory effects of Ang-(1-7) and alamandine in TNF-*α* induced by M(LPS+IFN-*γ*) were concentration-dependent with an optimal effect found at the concentration of 10^−7^ M for both peptides (Figures 4(e) and 4(f), respectively). Altogether, our results show that both peptides evoked anti-inflammatory responses in M(LPS+IFN-*γ*) macrophages while they may favor a phenotypic shift in the same subset toward a less inflammatory profile in a receptor- and concentration-dependent manner *in vitro*.

**Figure 5:**
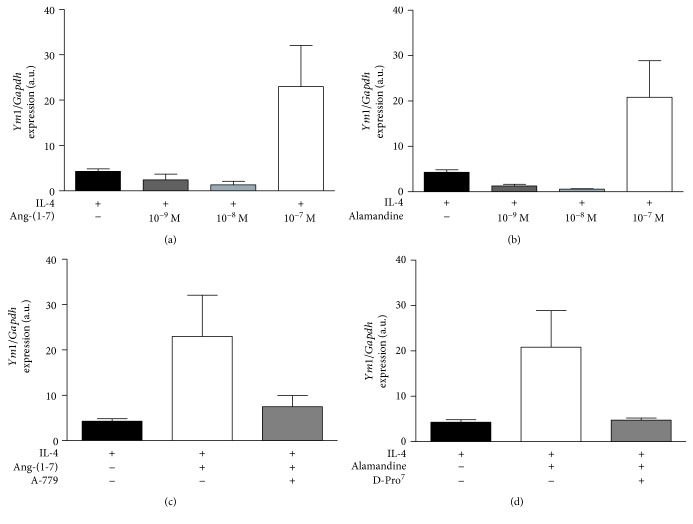
*In vitro* Ang-(1-7)- and alamandine-induced anti-inflammatory response in M(IL-4) macrophages. Real-time PCR analysis of (a) Ang-(1-7)- and (b) alamandine-induced YM1 mRNA levels in M(IL-4) macrophages at different concentrations. Analysis of YM1 expression levels in M(IL-4) cells (black bar) after administration of (c) Ang-(1-7) (10^−7^ M) and Ang-(1-7)+A779 (A779; 10^−6^ M) or (d) alamandine (10^−7^ M) and alamandine+D-Pro^7^ (D-Pro^7^; 10^−6^ M). Results were directly obtained by one-way ANOVA or by the nonparametric Kruskal-Wallis test after using logarithmic transformation and are expressed as the mean ± SEM of *n* = 4 independent experiments.

Next, we performed similar experiments in M(IL-4) macrophages. We observed that only the 10^−7^ M concentration of both Ang-(1-7) and alamandine (Figures 5(a) and 5(b), respectively) was able to induce an increase in YM1 expression, which had a trend to decrease after the administration of A-779 or D-Pro^7^, respectively (Figures 5(c) and 5(d)). Taken together, our data shows that Ang-(1-7) and alamandine may potentiate the IL-4-induced expression of YM1 in M2 macrophages.

### 3.3. Ang-(1-7) and Alamandine Promote the Resolution of LPS-Induced Pleurisy by Decreasing the Number of Neutrophils and the Frequency of M1 Inflammatory Macrophages

**Figure 6:**
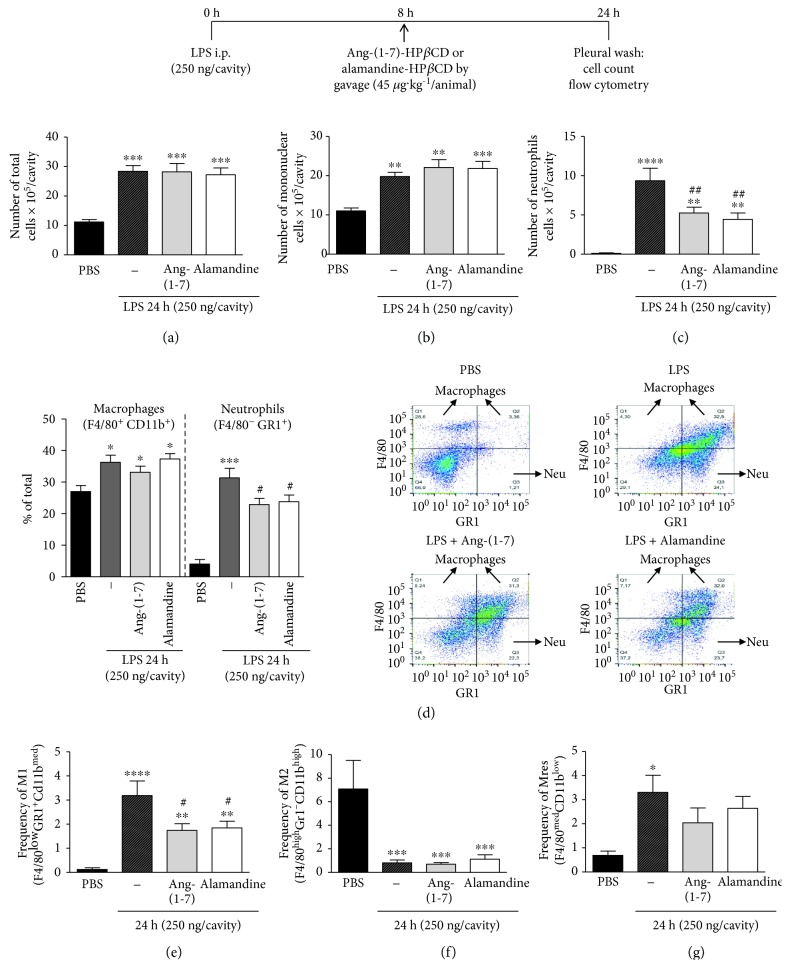
*In vivo* effects of Ang-(1-7) and alamandine treatments on the number of total and elicited cells and macrophage profile in the pleura during pleurisy. Analysis of the numbers of (a) total cells, (b) mononuclear cells, and (c) neutrophils by morphological counting from cytospin slides or by frequency of macrophages (F4/80^+^CD11b^+^) and neutrophils (GR1^+^F4/80^−^) by flow cytometry (d) as well as the frequencies of (e) M1, (f) M2, and (g) Mres macrophages in the pleural wash by flow cytometry in nontreated (PBS, *n* = 6, black bars) or LPS-treated animals (*n* = 7), and in the presence of LPS in treated animals with Ang-(1-7)-HP*β*CD (*n* = 8) or alamandine-HP*β*CD (*n* = 8). Representative dot plots are shown in (d). Results were directly obtained by one-way ANOVA or after logarithmic transformation (f only) and are expressed as the mean ± SEM. ^∗∗∗∗^ *P* < 0.0001, compared to PBS; ^∗∗∗^ *P* < 0.001, compared to PBS; ^∗∗,##^ *P* < 0.01, compared to PBS and LPS treatment only, respectively; ^∗,#^ *P* < 0.05, compared to PBS and LPS treatment only, respectively.

To evaluate *in vivo*, our *in vitro* findings, we tested the effects of Ang-(1-7) and alamandine on LPS-induced pleurisy in mice, a well-established model of acute inflammation [23, 33, 34, 38]. In this model, the intrapleural injection of LPS induces a time-dependent influx of leukocytes into the pleural cavity, which is characterized by early neutrophilic infiltration, with resolution at 48 h, when neutrophils are scarce and the number of mononuclear cells is maximal [33, 34]. In the present study, 8 h after LPS injection (corresponding to the peak of the neutrophilic infiltration), the animals were treated with either PBS, Ang-(1-7), or alamandine (both at 45 *μ*g·kg^−1^) and euthanized 24 h after LPS injection. Pleural wash contents were harvested and analyzed. As expected, LPS injection induced cell migration into the pleura and either Ang-(1-7) or alamandine was able to modify the numbers of total leukocytes (Figure 6(a)). Differential cell counting allowed the observation of intensified mononuclear cell migration at the 24 h time point as expected, but Ang-(1-7) and alamandine had no effects on that parameter (Figure 6(b)). Interestingly, both peptides were able to decrease the numbers of neutrophils into the pleura (Figure 6(c)). The analysis of populations frequencies by flow cytometry using known membrane markers to distinguish between macrophages (F4/80^+^CD11b^+^) and neutrophils (F4/80^−^GR1^+^) revealed similar findings to cytospin counting (Figure 6(d), with the respective representative dot plots). These results suggest that Ang-(1-7) and alamandine promote the resolution of LPS-induced inflammation, since both peptides decreased neutrophil numbers without modifying the numbers of mononuclear cells, normally involved in the clearance of the apoptotic neutrophils [39].

Previous studies from our group have characterized the macrophage phenotype in distinct phases of LPS-induced pleurisy. In PBS-injected mice, there is a predominance of M2 macrophages without proinflammatory macrophages. At the peak of LPS-induced pleurisy, there are mainly proinflammatory macrophages in the cavity, while at the resolving phase macrophages with anti-inflammatory and resolving phenotypes, there are predominant populations [23]. In the present study, we evaluated if the administration of Ang-(1-7) or alamandine at the peak of inflammation, when the inflammatory macrophages are predominant, could be able to modify the macrophage phenotype at the 24 h time point. Therefore, the frequency of M1 (F4/80^low^Gr1^+^Cd11b^med^), M2 (F4/80^high^Gr1^–^Cd11b^high^), and Mres (F4/80^med^Cd11b^low^) macrophage phenotypes in the pleural fluid was analyzed by flow cytometry using known markers for each subset (see supplementary Figure S3 for gating strategy). The administration of Ang-(1-7) or alamandine was able to reduce the increased LPS-induced frequency of M1 in the pleura (Figure 6(e)), as well as its numbers (data not shown). However, neither of them altered M2 and Mres frequencies (Figures 6(f) and 6(g)). Interestingly, we have found the same results in terms of the number of each macrophage phenotypes (data not shown). These results demonstrate that Ang-(1-7) and alamandine seem to have a predominant effect in M1 macrophages, resembling these data obtained *in vitro* by using a combination of LPS+IFN-*γ*.

## 4. Discussion

The role of the protective RAS axis, ACE2/Ang-(1-7)/Mas receptor, has been demonstrated in diverse *in vivo* models of experimental inflammation [2, 20, 40, 41]. However, there are few studies showing the direct participation of these protective RAS components in the distinct macrophage phenotypes. Souza and Costa-Neto et al. have previously shown the increased expression of Mas receptor transcript in naive mouse peritoneal macrophages after LPS stimulation [19]. The maximum peak of expression was observed 3 h after LPS incubation, and it was subsequently decreased at the 12 h. More recently, Hammer et al. demonstrated that the Mas receptor is expressed at both transcript and protein levels in distinct macrophage subsets. The Mas receptor transcript and protein were detected in M0, M(LPS+IFN-*γ*), and M(IL-4+IL-13) macrophages, with no significant differences after the 48 h period of stimulation [4]. In line with their work, we did not find differences in the Mas receptor expression among the distinct macrophage subtypes after 4 h stimulation *in vitro*, suggesting that the expression of the Mas receptor in macrophage is not influenced by cell culture duration.

Regarding the expression of the MrgD receptor, Habiyakare et al. showed a positive immunoreactivity for the anti-MrgD antibody in macrophage-containing atherosclerotic lesions from a rabbit aorta [42]. Yet in their serial section immunohistochemistry technique, the detection of anti-MrgD did not colocalize with RAM-1-positive cells, a macrophage marker [42]. Therefore, to the best of our knowledge, we are the first to show the expression of the mRNA and protein MrgD receptor in murine macrophages. In the present study, similarly to the Mas receptor, the expression of MrgD was not affected by the polarization state of macrophages *in vitro*. Nevertheless, it should be noted that macrophages also express other RAS components, having the machinery to metabolize multiple peptides. For instance, Rutkowska-Zapała et al. showed that the expression of ACE1 and ACE2 and the concentration of Ang II, Ang-(1-9), and Ang-(1-7) are differentially expressed among the naturally occurring human blood macrophage subpopulations [43]. While classical circulating macrophages (CD14^++^CD16^−^) presented high levels of Ang II, nonclassical macrophages (CD14^+^CD16^++^) presented an abundant Ang-(1-7)/Ang II ratio. Therefore, further studies are needed to evaluate the intracellular concentration of Ang-(1-7) and alamandine in murine bone marrow-derived M(LPS+IFN-*γ*) and M(IL-4)-polarized macrophages.

A remarkable feature of macrophages is their plasticity, which allows them to reprogram their functional states and phenotypes to adapt to their microenvironment [13, 44]. Considering that M0 macrophages can be promptly activated into any subset of the macrophage phenotype spectrum [45], we analyzed the influence of Ang-(1-7) and alamandine on resting cells. The results showed for the first time that Ang-(1-7) and alamandine per se did not affect the biochemical profile of M0 macrophages *in vitro* (Figure 3), although it is known that the RAS is crucial to macrophage maturation and function [46, 47]. In agreement, there is no modification of the expression of M2 (arginase-1 and MRC1) and M1 (iNOS) markers after bone marrow-derived macrophage incubation with Ang-(1-7) and alamandine for 24 h in culture (Figure S2). The ability of macrophages to switch from one phenotypic extreme to another, from M1 to M2 and vice versa, has been studied under different stimuli, from tissue injuries to a high-fat diet [48–51]. However, there is no previous report on the effects of Ang-(1-7) and alamandine on such plasticity. In the present study, we showed that both peptides induced an increase in YM1 and FIZZ1 gene expressions, important markers of M(IL-4) subset. Interestingly, this occurs simultaneously to the decreased gene expression of a set of proinflammatory cytokines. Indeed, both peptides increase the YM1 expression induced by IL-4, suggesting a cooperation between IL-4 and these peptides to promote the expression of this M2 marker. These data suggest that the actions of Ang-(1-7) and alamandine may be dual: skewing macrophages of their proinflammatory phenotype and promoting an anti-inflammatory phenotype in cooperation with IL-4.

Recent *in vitro* studies revealed the participation of an Src kinase family and the regulation of the phosphorylation levels of the Lyn kinase in the anti-inflammatory effects of Ang-(1-7) in LPS-induced TNF-*α* and IL-6 gene expression in macrophages [14, 19]. In addition, the administration of AVE0991, an analog of Ang-(1-7), decreased the expression of the same markers in M(LPS) macrophages, without affecting the expression of iNOS, IL-1*β*, and CCL2 transcripts [20]. In the present work, we corroborate previous findings that Ang-(1-7) evokes a decrease in the expression of these transcripts in M(LPS+IFN-*γ*) macrophages [19, 20, 52]. These effects on gene expression were inhibited by the Mas receptor antagonist, A-779. Intriguingly, A-779 was unable to reverse the diminished expression of IL-1*β* induced by Ang-(1-7). Indeed, previous data from Skiba et al. demonstrated that A-779 antagonism on the Mas receptor is enhanced when used at higher concentrations following treatment with AVE0991 [20]. This could explain why A-779 showed no significant effect on Ang-(1-7)-reduced IL-1*β* expression; nevertheless, we cannot exclude the participation of other intracellular mechanisms in this process. In agreement, previous studies using macrophages derived from Mas-deficient mice showed higher gene expression levels in two M(LPS+IFN-*γ*) markers, CCL2 and TNF-*α*, with no significant differences in iNOS, IL-6, and IL-1*β* gene expression when compared to macrophages from wild-type (WT) mice [4].

Regarding the M(IL-4) phenotype, AVE0991 in addition to M(IL-4+IL-13) macrophages increased the expression of the YM1 transcript and the cell surface marker MRC1; however, it did not affect other anti-inflammatory transcripts such as arginase-1, FIZZ1, MRC1, macrophage galactose N-acetylgalactosamine-specific lectin 2 (MGL2), and signaling lymphocytic activation molecule 1 (SLAMF1), neither the cell surface markers, cluster of differentiation 14 (CD14), nor major histocompatibility complex class II (MHCII) [4]. In the present work, the mRNA expression of YM1 was increased after treatment with Ang-(1-7) or alamandine. The effects of both peptides exhibited an inhibitory trend upon treatment with the receptor antagonists; however, it did not reach a statistical significance. Of note, Hammer et al. showed that a wide array of M(IL-4+IL-13) gene markers was downregulated in Mas-deficient macrophages compared to WT controls [4], suggesting the existence of a Mas receptor-dependent gene expression modulation.

In a similar way to Ang-(1-7), we have shown, for the first time, that alamandine decreases the proinflammatory responses of M(LPS+IFN-*γ*) macrophages, by downregulating the expression of TNF-*α*, CCL2, and IL-1*β*. In addition, we demonstrated for the first time that alamandine is also able to increase the expression of transcripts for M(IL-4) markers in the M(LPS+IFN-*γ*) phenotype *in vitro*. This reprogramming of M(LPS+IFN-*γ*) macrophage towards a less inflammatory and more proresolving phenotype naturally occurs during an acute inflammatory process as part of its resolution [39]. Thus, the ability of alamandine to induce macrophage plasticity could be considered a new therapeutic approach against autoimmune and chronic inflammatory diseases. Interestingly, in an *in vitro* model of LPS-induced inflammation and autophagy on neonatal rat cardiomyocytes exposed to LPS, the protective cellular effects of alamandine were associated to an inhibition of MAPK activation [53]. In addition, using a different proinflammatory stimulus, Ang II, Jesus and colleagues showed that the antihypertrophic effects of alamandine in neonatal rat cardiomyocytes are mediated by nitric oxide production and AMPK phosphorylation [54]. Therefore, further studies are needed to elucidate the underlying mechanism involved in alamandine-induced macrophage phenotype shift *in vitro*.

One of the main features of acute inflammation is the infiltration of neutrophils and their clearance by efferocytosis during resolution [39]. Previous works from our group showed that Ang-(1-7) and AVE0991 decrease the number of these cells via the Mas receptor in a murine model of adjuvant-induced arthritis [6, 55]. More specifically, Barroso et al. [6] have demonstrated in a mouse model of antigen-induced arthritis that therapeutic treatment with Ang-(1-7) at the peak of inflammation (the same way we performed here) decreases the number of neutrophils, a hallmark of resolution. Such an effect on neutrophil numbers caused by Ang-(1-7) was mirrored by increased apoptosis of these cells followed by macrophage efferocytosis [6]. In the present study, this peptide has a double effect: skewed M1 macrophages towards M2-like subset, which is known to have a higher efferocytic ability [56, 57], at the same time that induces a change in the gene expression profile towards a less inflammatory phenotype. Moreover, alamandine and Ang-(1-7) given at the peak of LPS-induced inflammation also induced the resolution of inflammation by decreasing the number of neutrophils. Such an effect of these peptides was probably due to an apoptosis-induced effect, as previously showed by Barroso et al. [6]. Therefore, the present study shows that similar to Ang-(1-7), alamandine has also anti-inflammatory *in vitro* and proresolving *in vivo* effects, reinforcing the need of additional investigations into its underlying mechanisms.

In animal models of pleurisy, both resident and elicited macrophages are activated to the M1 phenotype, which is responsible for further amplification of the inflammatory response [58]. As the immune response evolves, efferocytosis and environmental clues may reprogram macrophages towards the M2 phenotype [6, 34, 45], which is highly efferocytic and is responsible for phagocyting apoptotic neutrophils [6, 59], and producing considerable amounts of anti-inflammatory and tissue repair-related molecules [44]. In turn, M2 macrophages may switch to another phenotype, Mres, that partly resembles its predecessor and acts by favoring its resolution [37, 60, 61]. In addition, in the present study, we observed that both Ang-(1-7) and alamandine-treated animals presented reduced M1 macrophage frequencies, without affecting M2 or Mres cells *in vivo*. These *in vivo* findings corroborate our *in vitro* data, which shows that Ang-(1-7), via the Mas receptor, and alamandine, via the MrgD receptor, decrease the inflammatory responses of M(LPS+IFN-*γ*) macrophages and modulates M(IL-4) cells to a less extent. Therefore, treatments with either Ang-(1-7) or alamandine seem to orientate M1 macrophages towards an anti-inflammatory reprogramming, leading to a less inflammatory and more proresolving M2 phenotype at a longer term.

## 5. Conclusions

In summary, we have examined the effects of the two major protective RAS peptides, Ang-(1-7) and alamandine, on macrophage polarization *in vitro* and *in vivo*. We showed that both peptides act in M(LPS+IFN-*γ*) macrophages, reducing their inflammatory phenotype and increasing M(IL-4) markers *in vitro*. Ang-(1-7) and alamandine also decreased neutrophil numbers and M1 frequency in the pleural cavity during pleurisy *in vivo*, without influencing other macrophage populations over the investigated period of time. Further investigations are necessary to better understand the microenvironmental dynamics between RAS peptides and macrophages and therefore advancing on the potential use of these peptides as therapeutic targets for inflammatory diseases.

## Data Availability

The data used to support the findings of this study are available from the corresponding author upon request.

## Supplementary Materials

Supplementary 1
*In vitro* characterization of M(LPS+IFN-*γ*) and M(IL-4) macrophages. Real-time PCR analysis of expression levels of (a) TNF-*α*, (b) CCL2, (c) IL-1*β*, (d) YM1, (e) FIZZ1, and (f) MRC1 in resting (M0), M(LPS+IFN-*γ*)-polarized (LPS; 1 *μ*g/mL and IFN-*γ*; 20 ng/mL), and M(IL-4)-polarized (IL-4; 20 ng/m) macrophages. M(LPS+IFN-*γ*) markers: CCL2, TNF-*α*, and IL-1*β*; M(IL-4) markers: YM1, FIZZ1, and MRC1. Results were obtained by the nonparametric Kruskal-Wallis test and are expressed as the mean ± SEM of *n* = 4 independent experiments. ^∗∗∗^ *P* < 0.001, compared to M0; ^###^ *P* < 0.001, compared to M (IL-4); ^&&&^ *P* < 0.001, compared to M(LPS+IFN-*γ*).

Supplementary 2 Effect of Ang-(1-7) and alamandine on M0 macrophages. Bone marrow was isolated and differentiated to bone marrow-derived macrophages (BMDMs). The treatment protocol (24 h) was first standardized by the *in vitro* polarization of M0 macrophages towards M(LPS+IFN-*γ*) (a) or M(IL-4) (b and c). BMDMs were treated with either Ang-(1-7) or alamandine (10^−7^ M), and the mRNA of iNOS (a and d), MRC1 (b and e), and arginase-1 (c and f) were analyzed by real-time PCR. Results were obtained by a *t*-test (a–c) and one-way ANOVA (d–f) and are expressed as the mean ± SEM of *n* = 4 independent experiments. ^∗∗∗^ *P* < 0.001, compared to M0.

Supplementary 3 Gating strategy for the evaluation of macrophage populations in the pleurisy model. Leukocytes recovered from pleural cavity were stained with specific antibodies and analyzed by flow cytometry. Macrophage populations were defined according to F4/80, GR1 (Ly6G/Ly6C), and CD11b expression. Cells selected in the SSC × FSC gates (first dot plot) were analyzed for F4/80 and GR1 expression (second dot plot). F4/80^+^ cells were further analyzed for the intensity of F4/80^med^ population and then evaluated for CD11b^low^ cells, considered Mres (first row). M2 macrophages were defined by F4/80^+^GR1^−^ population and further analyzed for the intensity of F4/80^high^ expression followed by CD11b^high^ (second row). Finally, the F4/80^+^GR1^+^ population was analyzed for the intensity of F4/80^low^ population followed by CD11b^med^ expression to characterize the M1 population (third row).

Supplementary 4 Mouse primers used for quantitative real-time PCR.
