# Supplementary material for: Angiotensin-(1-7) and Alamandine Promote Anti-inflammatory Response in Macrophages In Vitro and In Vivo
Source: Mediators Inflamm. 2019 Feb 21;2019:2401081. doi: 10.1155/2019/2401081 (PMC6409041; doi:10.1155/2019/2401081)
Supplement: Supplementary 1 — In vitro characterization of M(LPS+IFN-γ) and M(IL-4) macrophages. Real-time PCR analysis of expression levels of (a) TNF-α, (b) CCL2, (c) IL-1β, (d) YM1, (e) FIZZ1, and (f) MRC1 in resting (M0), M(LPS+IFN-γ)-polarized (LPS; 1 μg/mL and IFN-γ; 20 ng/mL), and M(IL-4)-polarized (IL-4; 20 ng/m) macrophages. M(LPS+IFN-γ) markers: CCL2, TNF-α, and IL-1β; M(IL-4) markers: YM1, FIZZ1, and MRC1. Results were obtained by the nonparametric Kruskal-Wallis test and are expressed as the mean ± SEM of n = 4 independent experiments. ∗∗∗ P < 0.001, compared to M0; ### P < 0.001, compared to M (IL-4); &&& P < 0.001, compared to M(LPS+IFN-γ). [file 2401081.f1.pptx]

## Slide 1
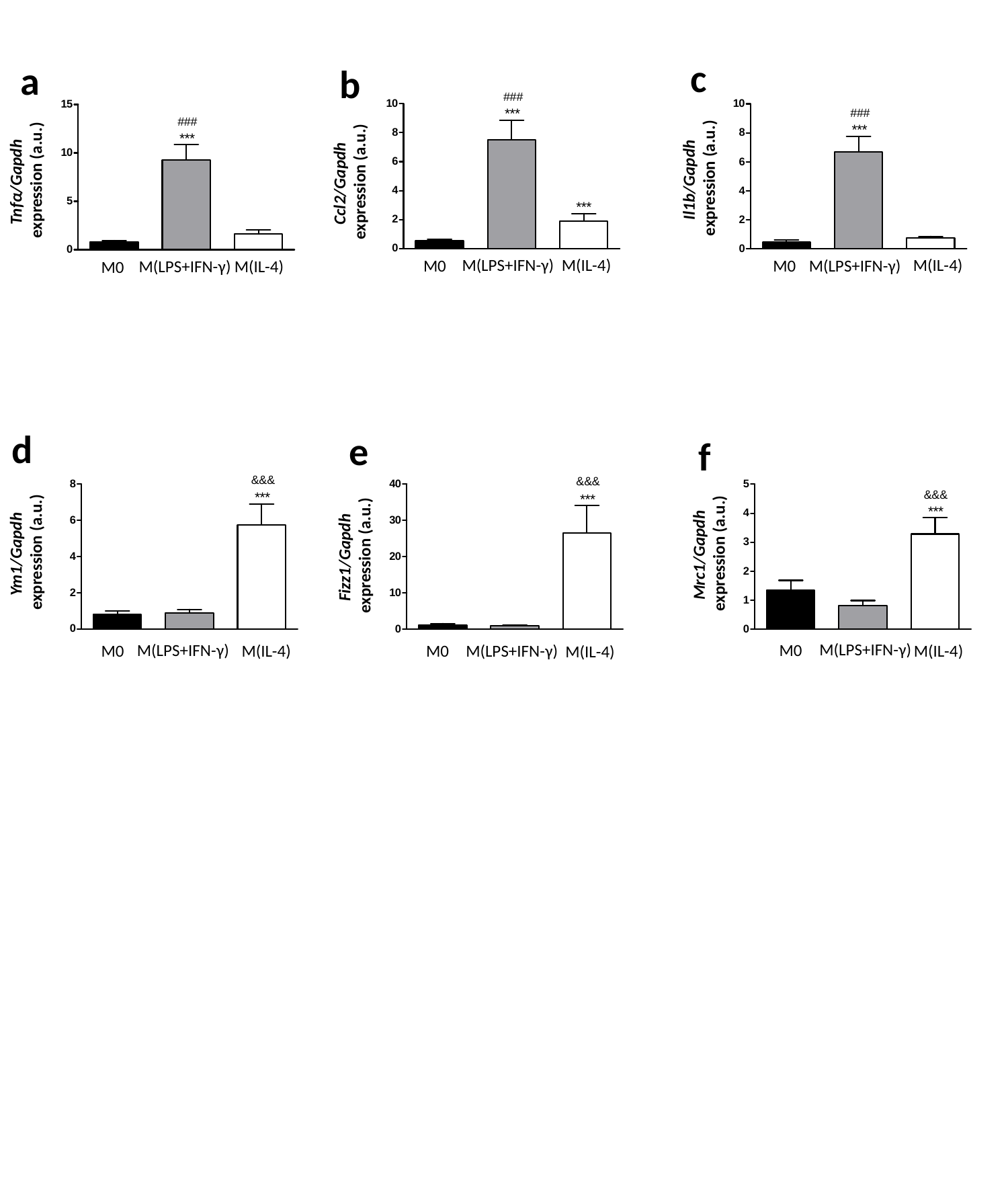

c
a
b
Il1b/Gapdh
 expression (a.u.)
Tnfα/Gapdh
 expression (a.u.)
Ccl2/Gapdh
 expression (a.u.)
M(LPS+IFN-γ)
M(IL-4)
M(IL-4)
M0
M0
M(LPS+IFN-γ)
M(IL-4)
M(LPS+IFN-γ)
M0
d
e
f
Ym1/Gapdh
 expression (a.u.)
Mrc1/Gapdh
 expression (a.u.)
Fizz1/Gapdh
 expression (a.u.)
M(LPS+IFN-γ)
M0
M(LPS+IFN-γ)
M(IL-4)
M0
M0
M(LPS+IFN-γ)
M(IL-4)
M(IL-4)
