# Supplementary material for: Angiotensin-(1-7) and Alamandine Promote Anti-inflammatory Response in Macrophages In Vitro and In Vivo
Source: Mediators Inflamm. 2019 Feb 21;2019:2401081. doi: 10.1155/2019/2401081 (PMC6409041; doi:10.1155/2019/2401081)
Supplement: Supplementary 2 — Effect of Ang-(1-7) and alamandine on M0 macrophages. Bone marrow was isolated and differentiated to bone marrow-derived macrophages (BMDMs). The treatment protocol (24 h) was first standardized by the in vitro polarization of M0 macrophages towards M(LPS+IFN-γ) (a) or M(IL-4) (b and c). BMDMs were treated with either Ang-(1-7) or alamandine (10−7 M), and the mRNA of iNOS (a and d), MRC1 (b and e), and arginase-1 (c and f) were analyzed by real-time PCR. Results were obtained by a t-test (a–c) and one-way ANOVA (d–f) and are expressed as the mean ± SEM of n = 4 independent experiments. ∗∗∗ P < 0.001, compared to M0. [file 2401081.f2.pptx]

## Slide 1
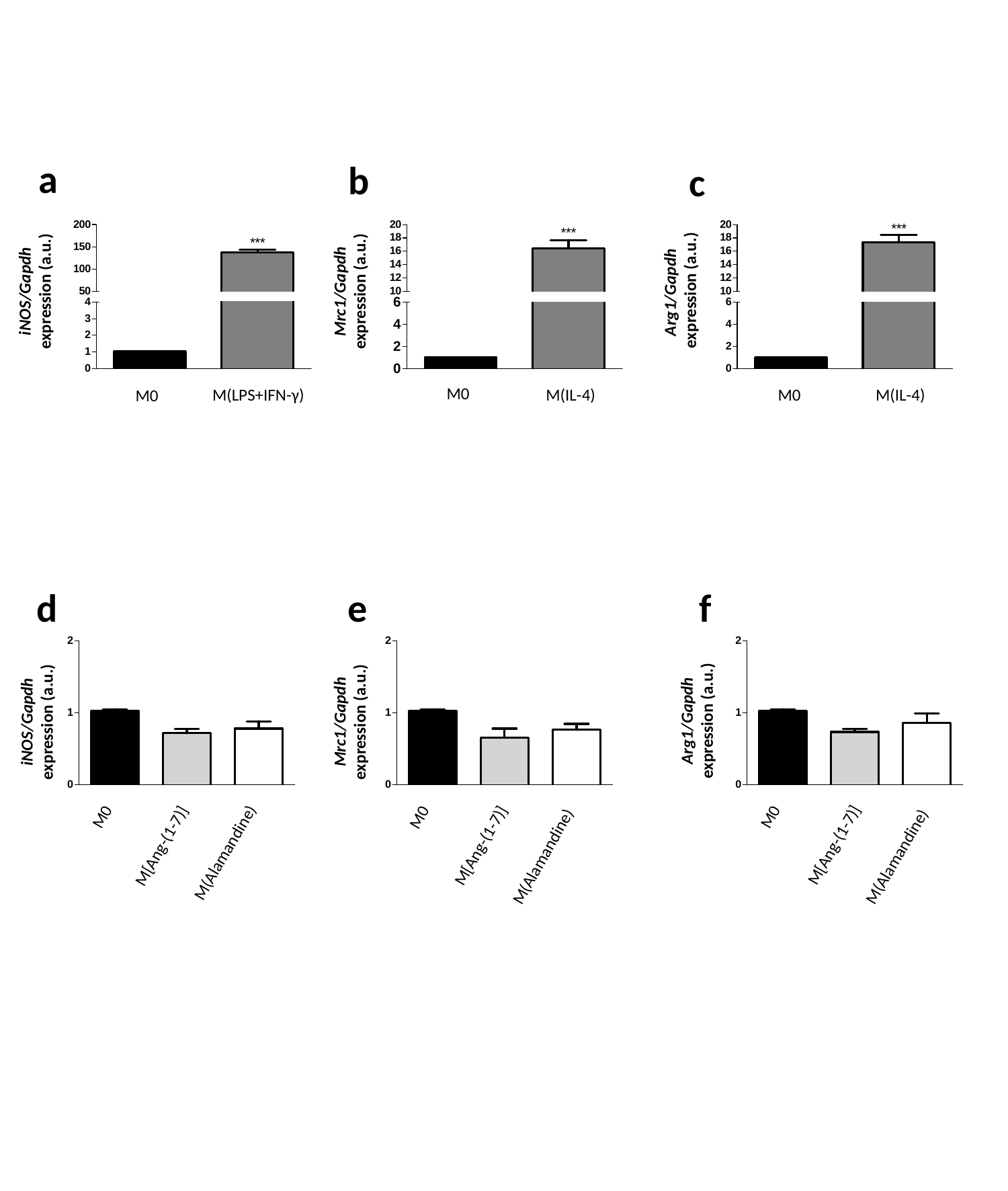

a
b
c
Arg1/Gapdh expression (a.u.)
iNOS/Gapdh expression (a.u.)
Mrc1/Gapdh expression (a.u.)
M0
M(LPS+IFN-γ)
M(IL-4)
M(IL-4)
M0
M0
d
e
f
Arg1/Gapdh expression (a.u.)
iNOS/Gapdh expression (a.u.)
Mrc1/Gapdh expression (a.u.)
M(Alamandine)
M(Alamandine)
M[Ang-(1-7)]
M(Alamandine)
M[Ang-(1-7)]
M[Ang-(1-7)]
M0
M0
M0
