# Supplementary figures and images for: Angiotensin-(1-7) and Alamandine Promote Anti-inflammatory Response in Macrophages In Vitro and In Vivo
Source: Mediators Inflamm. 2019 Feb 21;2019:2401081. doi: 10.1155/2019/2401081 (PMC6409041; doi:10.1155/2019/2401081)

## Slide 1
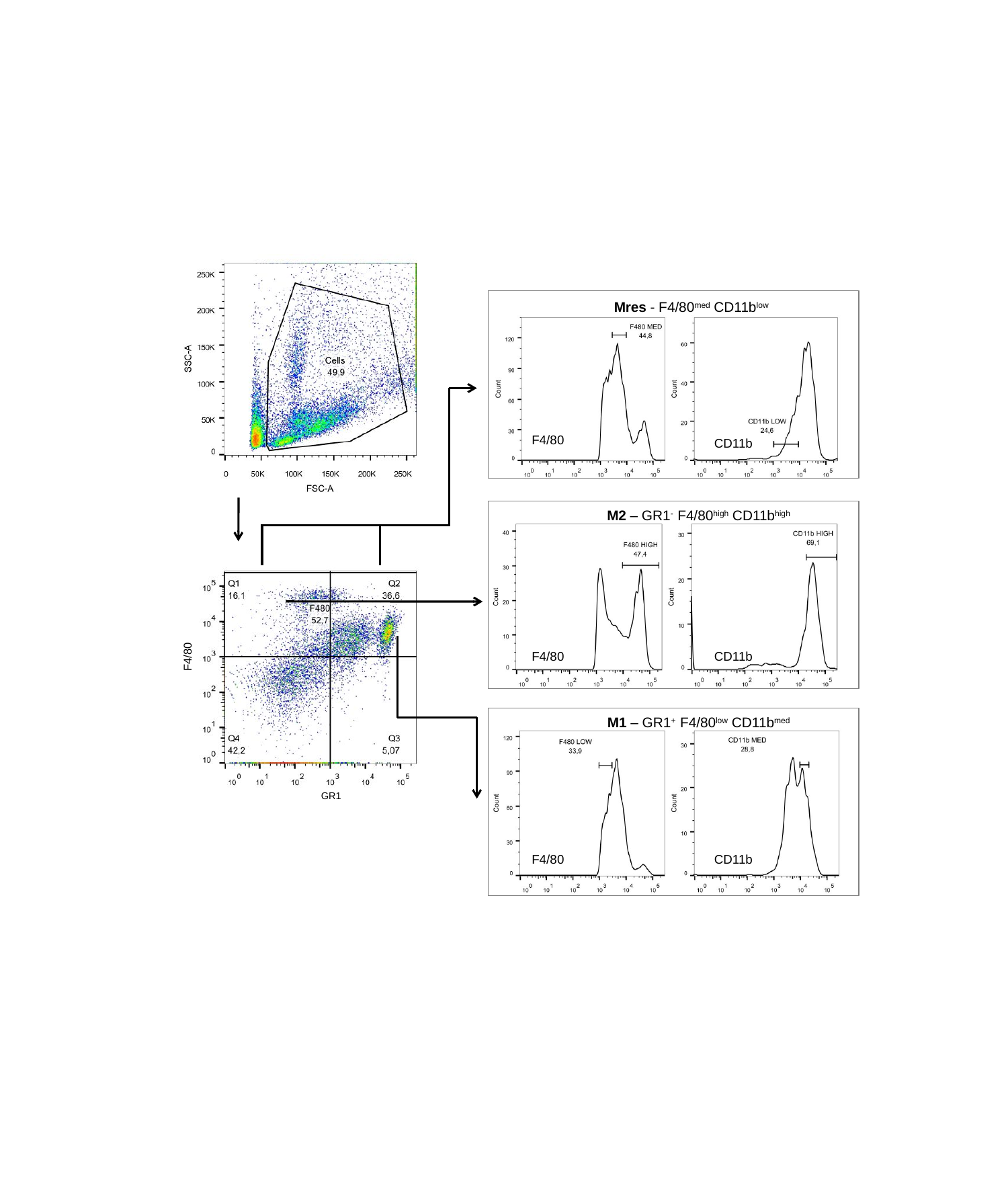

Mres - F4/80med CD11blow
F4/80
CD11b
M2 – GR1- F4/80high CD11bhigh
F4/80
CD11b
F4/80
GR1
M1 – GR1+ F4/80low CD11bmed
F4/80
CD11b

Supplement: Supplementary 3 — Gating strategy for the evaluation of macrophage populations in the pleurisy model. Leukocytes recovered from pleural cavity were stained with specific antibodies and analyzed by flow cytometry. Macrophage populations were defined according to F4/80, GR1 (Ly6G/Ly6C), and CD11b expression. Cells selected in the SSC × FSC gates (first dot plot) were analyzed for F4/80 and GR1 expression (second dot plot). F4/80+ cells were further analyzed for the intensity of F4/80med population and then evaluated for CD11blow cells, considered Mres (first row). M2 macrophages were defined by F4/80+GR1− population and further analyzed for the intensity of F4/80high expression followed by CD11bhigh (second row). Finally, the F4/80+GR1+ population was analyzed for the intensity of F4/80low population followed by CD11bmed expression to characterize the M1 population (third row). [file 2401081.f3.pptx]
