# Supplementary material for: Angiotensin-(1-7) and Alamandine Promote Anti-inflammatory Response in Macrophages In Vitro and In Vivo
Source: Mediators Inflamm. 2019 Feb 21;2019:2401081. doi: 10.1155/2019/2401081 (PMC6409041; doi:10.1155/2019/2401081)
Supplement: Supplementary 4 — Mouse primers used for quantitative real-time PCR. [file 2401081.f4.doc]

Table S1: Mouse primers used for quantitative real-time PCR

| **Gene** | **Function** | **Nucleotide sequence** |
| --- | --- | --- |
| ARG1^a^  ARG1^a^ | Foward  Reverse | 5'-TGACATCAACACTCCCCTGACAAC-3'  5'-GCCTTTTCTTCCTTCCCAGCAG-3' |
| CCL2  CCL2 | Foward  Reverse | 5’-TTAAAAACCTGGATCGGAACCAA -3’  5’-GCATTAGCTTCAGATTTACGGGT -3’ |
| FIZZ1^b^  FIZZ1^b^ | Foward  Reverse | 5’- CCCTCCACTGTAACGAAGACTC -3’  5’- CACACCCAGTAGCAGTCATCC -3’ |
| GAPDH  GAPDH | Foward  Reverse | 5’ - TGCGACTTCAACAGCAACTC -3’  5’ - ATGTAGGCCATGAGGTCCAC -3’ |
| IL-1β  IL-1β | Foward  Reverse | 5’- GCAACTGTTCCTGAACTCAACT -3’  5’- ATCTTTTGGGGTCCGTCAACT -3’ |
| iNOS^a^  iNOS^a^ | Foward  Reverse | 5’-AGCACTTTGGGTGACCACCAGGA-3’  5’-AGCTAAGTATTAGAGCGGCGGCA-3’ |
| Mas receptor | Foward | 5’- AGGGTGACTGACTGAGTTTGG -3’ |
| Mas receptor | Reverse | 5’- GAAGGTAAGAGGACAGGAGC -3’ |
| MRC1^b^  MRC1^b^ | Foward  Reverse | 5’- CATGAGGCTTCTCCTGCTTCTG -3’  5’- TTGCCGTCTGAACTGAGATGG -3’ |
| MrgD | Foward | 5’- TTTTCAGTGACATTCCTCGCC -3’ |
| MrgD | Reverse | 5’- GCACATAGACACAGAAGGGAGA -3’ |
| TNFα  TNFα | Foward  Reverse | 5’- CATCTTCTCAAAATTCGAGTGACAA -3’  5’- TGGGAGTAGACAAGGTACAACCC -3’ |
| YM1^d^  YM1^d^ | Foward  Reverse | 5’- GGGCATACCTTTATCCTGAG -3’  5’- CCACTGAAGTCATCCATGTC -3’ |

^a^ SUGIMOTO, M.A. et al. (2017). Plasmin and plasminogen induce macrophage reprogramming and regulate key steps of inflammation resolution via annexin A1. Blood 2017; v. 129, p.2896–2907.

^b^ KARO-ATAR, D. et al. (2013). Paired immunoglobulin-like receptor-B inhibits pulmonary fibrosis by supressing profibrogenic properties of alveolar macrophages. *Am J Respir Cell Mol Biol*, v. 48, n. 4, p. 456-464.

^c^ JUNG, U.J. et al. (2012). Fatty Acids Regulate Endothelial Lipase and Inflammatory Markers in Macrophages and in Mouse Aorta: A Role for PPARγ. *Arteriosclerosis, thrombosis, and vascular biology*, v. 32, n. 12, p. 2929–2937.

^d^ RAES, G. et al. (2002). FIZZ-1 e YM as tools to discriminate between differentially activated macrophages. *Dev immunol,* v. 9, n. 3, p. 151-159.

All other primer sequences were designed by the Laboratory of Hypertension (UFMG, Brazil).
